# Numerical Simulations of 3C-SiC High-Sensitivity Strain Meters

**DOI:** 10.3390/mi16090989

**Published:** 2025-08-28

**Authors:** Annamaria Muoio, Angela Garofalo, Sergio Sapienza, Francesco La Via

**Affiliations:** 1 CNR-IMM, Strada VIII 5, 95121 Catania, Italy; 2 Materials Science Department, Milano-Bicocca University, Via R. Cozzi 55, 20125 Milano, Italy; a.garofalo22@campus.unimib.it; 3 CNR-ISMN, Via Piero Gobetti 101, 40129 Bologna, Italy; sergio.sapienza@cnr.it

**Keywords:** COMSOL Multiphysics, loss factor matrix, anisotropic damping

## Abstract

In the simulation of 3C-SiC strain gauges in dynamic environment—particularly those involving vibrations and wave propagation—the accurate representation of energy dissipation is essential for reliable predictive modeling. This paper discusses the implementation of both isotropic and anisotropic damping models within COMSOL Multiphysics. In particular, it focuses on the use of an anisotropic loss factor, represented either as a scalar (η_S_) for isotropic cases or as a symmetric 6 × 6 loss factor matrix ($\eta_{D}$) for anisotropic dissipation. This formulation enables the directional dependence of damping behavior to be captured, which is particularly important in composite materials, layered media, and metamaterials where energy dissipation mechanisms vary with orientation. The paper also explores the numerical implications of using anisotropic damping, such as its influence on eigenfrequency solutions, frequency response functions, and transient dynamic simulations. Furthermore, it highlights how the inclusion of directional damping can improve the correlation between simulated and experimental results in scenarios where standard isotropic models fail to capture key physical behaviors.

## 1. Introduction

Dynamic systems are inherently dissipative; vibratory energy is gradually converted into heat or other forms of energy, leading to a reduction in oscillation amplitude. In finite element analysis (FEA) using COMSOL Multiphysics^®^ (Version 6.1) [1], this damping behavior can be modeled using conventional damping parameters as well as more sophisticated approaches, such as anisotropic loss factors [2]. The isotropic loss factor ($\eta_{s}$) assumes uniform energy loss in all directions, while anisotropic damping employs a symmetric 6 × 6 loss factor matrix ($\eta_{D}$), where only the components in the upper-triangular portion are independently defined. This distinction is critical for accurately simulating the directional dependencies observed in heterogeneous materials, such as composites and layered structures. Several mathematical frameworks exist to describe damping in structural dynamics. Typically, the damping-induced decay of vibration amplitudes is modeled as an exponential decrease over time. The phase information contained in a complex-valued mode shape can reveal local phase shifts, offering insight into spatial variations in damping behavior. In COMSOL Multiphysics, the damping matrix is assembled by combining various damping contributions, which are then incorporated into the finite element model to capture phenomena like mode shape distortions and resonant peak broadening. The flexibility of COMSOL Multiphysics allows users to define loss factors along different material orientations, thereby enhancing the predictive capability of numerical simulations. This capacity to represent anisotropic energy dissipation is particularly important in fields like aerospace engineering and seismic analysis, where the precise characterization of damping effects directly impacts performance reliability and safety. The accurate modeling of damping is not only essential for realistic dynamic simulations but also for improving the correlation with experimental data. Anisotropic damping is particularly relevant in fiber-reinforced composites and layered structures where energy dissipation varies with direction. Complex mode shapes allow for the identification of localized damping phenomena, while matrix-based damping formulations support frequency-dependent analyses.

## 2. Materials and Methods

**Figure 1:**
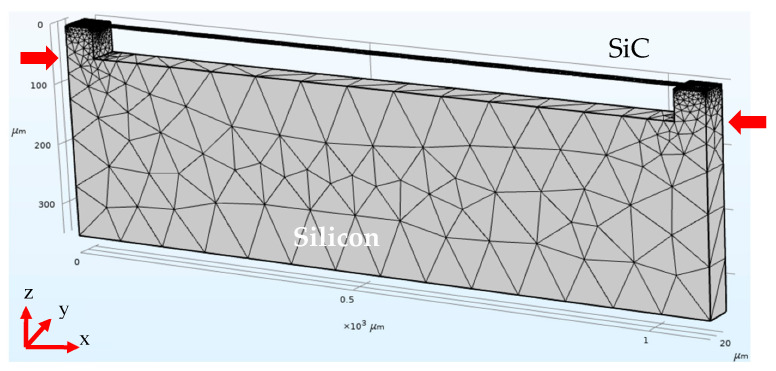
COMSOL simulated image of the sample: (111) 3C-SiC double-clamped beam grown on silicon substrate. The resulting mesh consisted of tetrahedral domain elements, triangular boundary elements, and edge elements. The red arrows, parallel to the *x* direction, indicate the force direction for the simulation of compressive/tensile stress.

The samples in this work are 3C-SiC double-clamped beam structures (Figure 1) previously fabricated and characterized at CNR-ISMN Bologna (Italy), with Novasic wafers from Zielinski in France [3]. These samples were originally fabricated for the investigation of the Model of Quality factors for (111) 3C-SiC resonators. All of the comprehensive descriptions of the epitaxial growth process [4,5], lithographic patterning, and etching steps used to fabricate the devices are available in the original publication [3].

The samples are (111) 3C-SiC film grown on silicon substrates, with a low doping concentration (<$10^{16\;}cm^{-3},$ NID—not intentionally doped), in a double-clamped beam configuration with a fixed width of 16 µm, lengths ranging from 200 to 1000 µm, and varying thicknesses. The complete list of samples is reported in Table 1.

### 2.1. Finite Element Formulation

The dynamic behavior of a structural system is described through partial differential equations (PDEs), which are discretized using the finite element method (FEM) to yield a system of matrix equations. In matrix form, the equations of motion for a discretized structure are written as

(1) $$M\;\cdot \;\ddot{u}+C\;\cdot \;\dot{u}+K\;\cdot u=f$$

where *M* is the mass matrix, *C* is the damping matrix, *K* is the stiffness matrix, u is the displacement vector, and *f* denotes the applied force vector. The damping matrix *C* can be formulated to include various damping contributions, with both isotropic and anisotropic components. The mass and stiffness matrices are determined through geometry and intrinsic material properties (i.e., density and Young’s modulus), whereas the damping matrix reflects the energy dissipation mechanisms. The damping matrix *C*, which captures energy dissipation mechanisms, can be formulated using various approaches, including Rayleigh (mass and stiffness proportional), modal, or complex-valued formulations. For isotropic materials, scalar loss factors are often sufficient, but, in the case of anisotropic or composite structures, C must be constructed to reflect directional dependencies, typically via a symmetric 6 × 6 loss factor matrix. This anisotropic formulation is particularly relevant for orthotropic layers and fiber-reinforced composites, where damping varies with material orientation [7,8]. Although the eigenvalues of the anisotropic loss factor matrix obtained from COMSOL do not numerically match those of the stiffness matrices reported in the literature for materials like 3C-SiC or 4H-SiC, this discrepancy is expected due to their fundamentally different physical interpretations. The stiffness matrix describes the material’s elastic response, while the loss factor matrix characterizes its energy dissipation behavior. However, both matrices are defined in the same Voigt space of strain components, and their eigenvectors represent principal deformation directions. The observed alignment between the principal directions of damping and elasticity suggests that the material exhibits directional mechanical behavior consistent with anisotropy. This justifies the adoption of an anisotropic damping model in our simulations, as it allows for a more accurate prediction of vibrational phenomena and eigenfrequencies, particularly in thin micro- and nano-scale films, where such effects become prominent.

### 2.2. Simulation Considerations in COMSOL Multiphysics

When performing structural dynamic analysis in COMSOL Multiphysics, it is essential to carefully consider the following aspects:

- Damping Representation: The choice between isotropic (scalar η_S_) and anisotropic (matrix $\eta_{D}$ ) damping models should be guided by the material system under investigation.
- Energy Dissipation: The simulation should capture not only the decay rate of vibratory energy but also the phase relationships within the mode shapes. These details are crucial for a comprehensive understanding of damping effects.
- Discretization Accuracy: Finite element discretization provides an approximation of the true solution of the PDEs governing the system’s behavior. Ensuring that the mesh is sufficiently refined is key to obtaining reliable and accurate results.
- Stress Analysis: In addition to damping effects, the distribution of internal stresses—both compressive and tensile—should be evaluated. For instance, when a bridge is deformed under load, the generated stress patterns offer valuable insight into the structural performance and possible failure mechanisms. (A representative stress image of a bridge can be included as part of the simulation outputs).

The choice between isotropic and anisotropic damping reflects not only material anisotropy but also the frequency-dependent nature of energy dissipation. Accurately capturing phase lag in mode shapes helps identify modal coupling and potential resonance risks [9,10].

To ensure the reproducibility and reference value of the simulation work, the mesh parameters were carefully defined, including the element type and the maximum and minimum element size. Particular attention was paid to the resolution near boundary regions and areas with expected high gradients in order to balance computational efficiency and solution accuracy. All mesh settings are documented to allow for consistent replication and validation of the numerical results. The resulting mesh consisted of 10,930 tetrahedral domain elements, 5276 triangular boundary elements, and 885 edge elements (Figure 1).

## 3. Results

**Figure 2:**
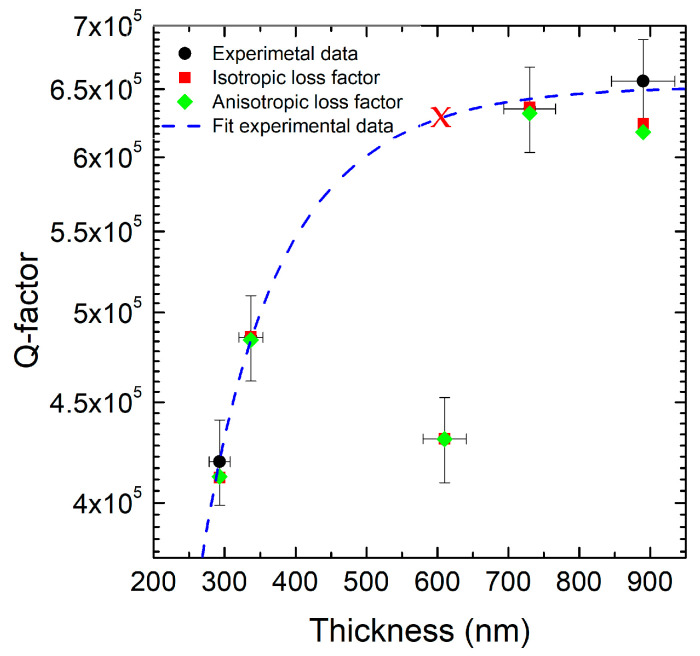
Plot of the Q-Factor as a function of the thickness of a material, expressed in nanometers (nm). The data represent three different conditions: experiment (black), isotropic loss factor simulation (red), and anisotropic loss factor simulation (green). The blue dashed line represents the theoretical fit of experimental data [3]. The red cross indicates the expected value of the experimental Q-Factor for a thickness of 610 nm.

The Q-Factor increases significantly with increasing thickness, especially between 500 and 700 nm (Figure 2). Beyond 700 nm, the Q-Factor stabilizes or shows a slight decrease. Using both models of isotropic and anisotropic dumping, it is possible to fit the experimental data with good accuracy. But, using this fitting, it is not possible to obtain with the same accuracy the resonance frequency for the different film thicknesses. The experimental values (black circles) range from ~4.05 × 10^5^ at 300 nm to ~6.60 × 10^5^ at 870 nm. The anisotropic loss factor model (green diamonds) shows excellent agreement, with errors below ±5.5% across all thicknesses. The isotropic model (red squares) deviates by up to +1.7% at 600 nm and has reduced accuracy in thicker films. These results quantitatively confirm that the anisotropic damping model more closely reproduces the measured energy dissipation behavior, particularly in the upper thickness range. The Q-Factor of the value at 610 nanometers deviates from the fit of the experimental values (lower by about 30%) due to the residual stress (in Figure 2, the red cross indicates the expected value around $6\times 10^{5}$).

**Figure 3:**
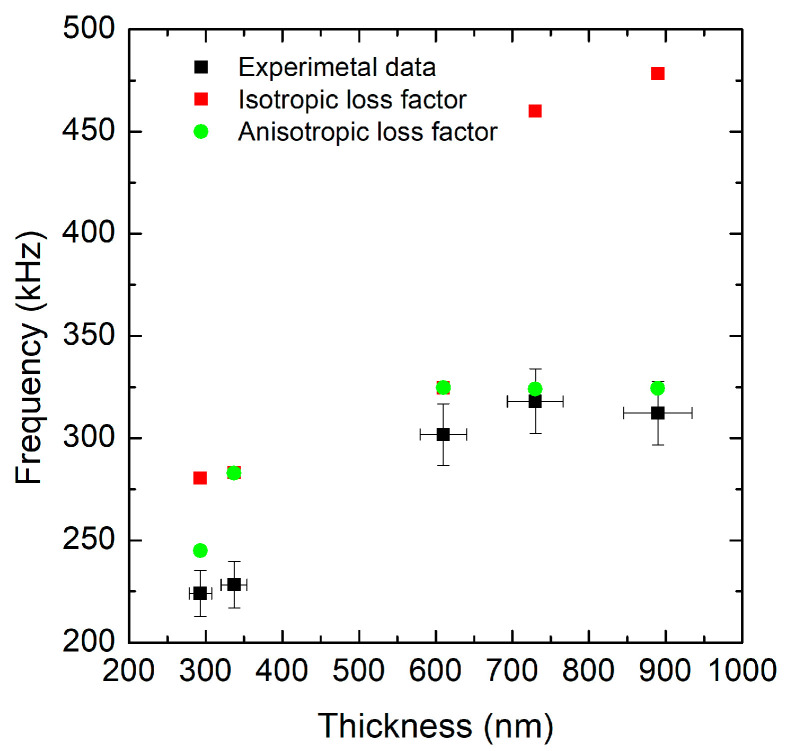
Red curve: COMSOL simulations considering the isotropic loss factor in the eigenfrequency analysis. Green curve (diamonds): COMSOL simulations considering the anisotropic loss factor in the eigenfrequency analysis. Black curve: Experimental data. A mesh convergence study was performed, confirming that the selected mesh parameters yield stable results with less than 1% variation in the first eigenfrequency. (Error bands: 5% for experimental resonance frequency and thickness).

In fact, the experimental data show an increase in frequency with thickness up to around 700 nm, followed by a slight decrease (Figure 3). The isotropic loss factor simulation overestimates the frequency for thinner films but significantly overestimates it for thicknesses above 600 nm, showing a sharp increase not observed experimentally. In contrast, the anisotropic loss factor simulation closely follows the experimental trend across the entire thickness range, with a larger estimate of the resonance frequency only for the thinner layers. The agreement between experimental data and the anisotropic model is clearly better than with the isotropic one. This suggests that the material exhibits anisotropic behavior and that including anisotropy in the loss factor is crucial for accurately predicting eigenfrequencies. The isotropic simulation lacks key directional mechanical effects or damping mechanisms, which likely become significant at higher thicknesses—hence the large overestimation above 600 nm. The drop in the experimental frequency after 700 nm might be due to additional real-world effects not captured by the simulations, such as tension changes in mechanical properties (i.e., density, Young’s modulus) and geometric effects (i.e., increased bending influence at greater thicknesses). The isotropic model systematically overestimates the frequency, with errors of up to +50%, while the anisotropic model shows a better fit with the experimental data.

**Figure 4:**
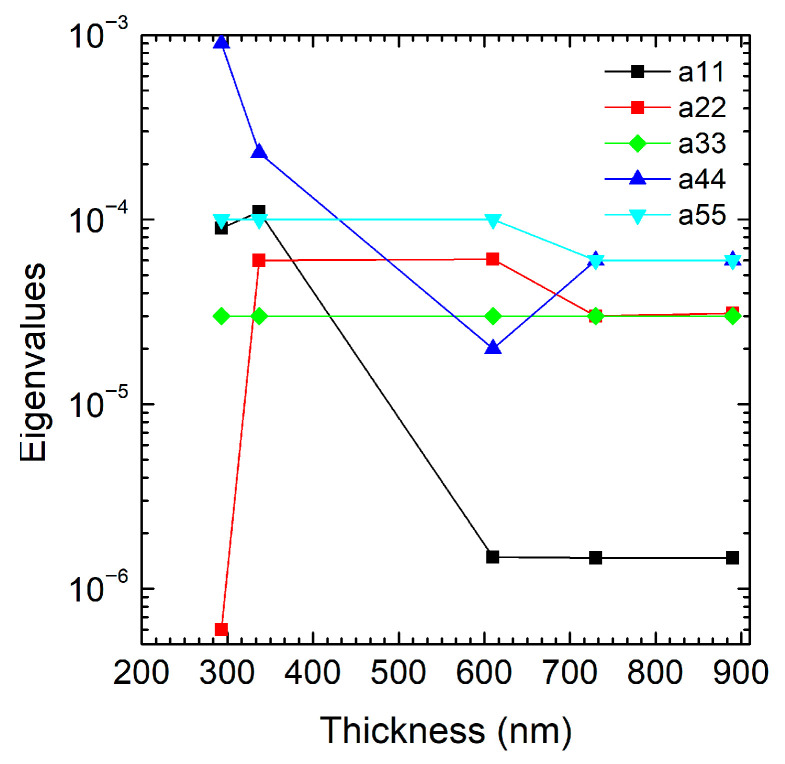
Plot of the eigenvalues as a function of the 3C-SiC film thickness (nm), distinguishing $a_{11}$ (black dots), $a_{22}$ (red dots), $a_{33}$ (green dots), $a_{44}$ (blue dots), and $a_{55}$ (cyan dots).

The loss factor matrix ($\eta_{D}$) describes how damping losses vary along different material directions. The eigenvalues (a_11_, a_22_, a_33_, a_44_, a_55_) represent the principal damping values along the main anisotropy axes. Specifically, a_11_, a_22_, and a_33_ correspond to the x, y, and z directions, respectively, while a_44_ and a_55_ are associated with the shear damping in the yz and *xz* planes. A summary of the eigenvalues, their associated directions, and the corresponding damping mechanisms is reported in Table 2. In Figure 4, a comparison between these eigenvalues shows how direction dependent the damping is (i.e., $a_{44}$ >> $a_{11}$ >> $a_{22}$ indicates strong damping along few directions). If the plot shows similar eigenvalues ($a_{11}$ ≈ $a_{22}$ ≈ $a_{33}$), the material is nearly isotropic in damping. If they differ significantly (i.e., $a_{11}$ > $a_{22}$, $a_{33}$), the material is highly anisotropic, with losses dominated along one direction (e.g., fibers in a composite). In our case, $a_{11}$ (black line) starts with relatively high values (~1.0 × 10^−4^), peaking around 400 nm; after 400 nm, the value drops sharply to nearly 0 and remains negligible for thicknesses above 600 nm, indicating that losses along direction *x* are suppressed as the thickness increases.

The damping along *x* is dominant in thinner films because under these conditions, the longitudinal direction is the main site of deformation and dissipation. For thicker films, the stiffness along *x* increases, which means less deformation and dissipation. The red line, $a_{22}$, sharply increases for the lower thickness, and above 300 nm it almost remains constant (~$10^{-5}$). The sharp growth for $a_{22}$ observed above 300 nm may signal a new regime, where losses in the *y* direction are increased, while for higher thickness the anisotropic losses are lower. The transverse direction is a thickness-dependent threshold, participating in dissipation when the flexural modes also involve the *y* axis. The green line, $a_{33}$, remains nearly constant over the entire thickness range (~2.5 × 10^−5^), suggesting low sensitivity to thickness variations above 300 nm. Furthermore,$\;a_{33}$ is the most stable, which could suggest that losses in direction *z* are less sensitive to thickness and generally minimal. The dissipation in the *z* direction is marginal. The blue line, $a_{44}$, decreases, reaching a minimum at 600 nm, and, after a slight increase, it stays constant. It then shows shear damping in the *yz* plane, which is initially significant, and then a slight decrease in thicker films due to the vibration modes’ shift from torsion/shear to pure bending. For the cyan line, $a_{55}$, no great variation in value can be observed in the entire range of film thickness; in the *xz* plane, the shear damping is thickness independent. The significant reduction in the $a_{ij}$ coefficients, particularly $a_{11}$, indicates lower anisotropic losses in the system, contributing to increased efficiency (higher Q-Factor).

In many practical applications, the inclusion of damping in eigenfrequency analyses is primarily aimed at estimating the attenuation levels of resonant modes. Although the effect of damping on the mode shapes and eigenfrequencies is often marginal, its accurate implementation is essential for predicting the performance of structures under dynamic loading conditions. The use of a matrix of anisotropic loss factors is particularly advantageous when dealing with systems in which energy dissipation is strongly dependent on material orientation during the propagation of elastic waves; this phenomenon is closely related to the attenuation tensor or the viscosity tensor, which represents the imaginary part of the complex elastic modulus. Compared to the Joffe model [11], this is a micromechanical approach used to predict the effective elastic properties of composite materials. The model considers the contributions of the fibers and matrix phases, including their volume fractions and orientations, which represents the real part of the elastic modulus. Below, Voigt notation was used to simplify symmetric tensors (like stress/strain) into vectors/matrices as

(2) $$C^{*}=C+iw\eta_{D}$$

where *C* and η $\in R^{6\times 6}$ are C = real part = conservative elastic matrix and $iw\eta_{D}=$ imaginary part = attenuation tensor matrix.

Compared to the Joffe model—which focuses on micromechanical predictions of effective elastic properties based on fiber–matrix interactions—the anisotropic damping model used in this study directly incorporates directional energy dissipation through a 6 × 6 loss factor matrix. While the Joffe model captures the real part of the elastic modulus, our approach targets the imaginary part, which governs attenuation and damping. This distinction allows for more accurate modeling of vibrational decay and resonance behavior in 3C-SiC structures. The anisotropic damping matrix shares conceptual similarities with the viscosity tensor model, often used in wave propagation studies. However, our implementation in COMSOL enables direct integration into FEM simulations, offering better control over directional damping and improved correlation with experimental data. The directional damping captured by the anisotropic model aligns with the known anisotropic stiffness of 3C-SiC, as reported by Thomsen [7] and Yang et al. [8]. The propagation of elastic waves in such materials is governed not only by stiffness tensors but also by attenuation tensors. Our model bridges this gap by incorporating both elastic and dissipative anisotropy, enabling more realistic simulation of wave behavior in MEMS resonators.

**Figure 5:**
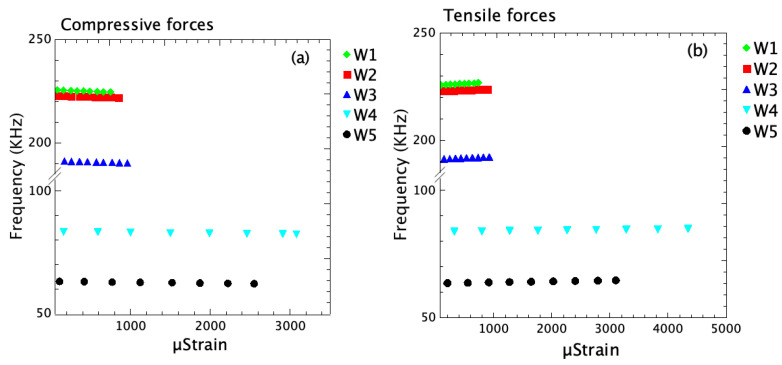
Relationship between μstrain and frequency (in kHz) under compressive force conditions, (**a**,**b**) tensile forces.

By applying compressive forces to the bridge and then producing a compressive strain in the structures, it is possible to simulate the decrease of the resonance frequency for the different samples. From these simulations, in Figure 5a, it is possible to observe that the bridges that have the highest resonance frequency ($w_{2}$) and the highest tensile intrinsic stress are less sensitive to the compressive forces and produce the lowest deformation of the bridges. Instead, the thinner bridges, which are also those ones with the lowest tensile stress, show a larger deformation under the same compressive forces.

The frequency decreases as compressive strain increases. Thicker beams show higher initial resonance frequencies and lower sensitivity to compressive strain. Thinner beams show greater frequency shifts, indicating higher mechanical compliance and lower intrinsic tensile stress. The plot in Figure 5b shows how the strain varies as a function of frequency under the application of tensile forces. Both $w_{4}$ and $w_{5}$ samples’ configurations [6] show a strong strain response to tensile forces, which is even more pronounced than under compressive forces. The $w_{5}$ shows the highest strain among all, suggesting strong mechanical resonance or greater tensile compliance at that frequency. The $w_{1}$, $w_{2}$, and $w_{3\;}$ series respond weakly under tension, with very small strain magnitudes. The overall strain under tensile loading is higher than in the compressive case for the same configurations ($w_{4}\;$ and $w_{5}$), indicating that the material or the structure is more compliant or deformable when pulled than when compressed [12]. The frequency increases with tensile strain and the response is more pronounced than in compression, suggesting that the material is more compliant in tension. The percentage errors between simulated and experimental values for each sample are summarized in Table 3. All values are within ±7%, indicating very good agreement. The anisotropic model used in the simulation appears to accurately capture the strain sensitivity trends across all samples.

The percentage errors between simulated and experimental values are all within ±7%, indicating very good agreement. The anisotropic model used in simulation appears to accurately capture the strain sensitivity trends across all samples.

**Figure 6:**
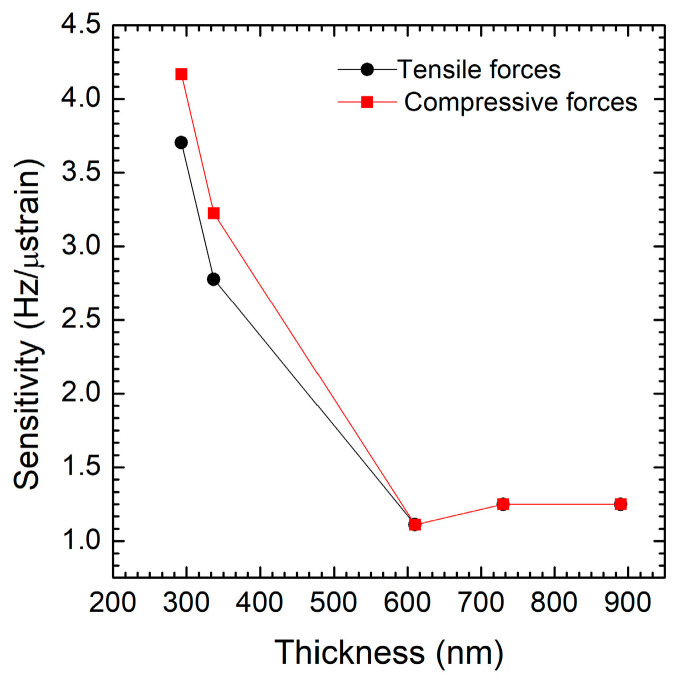
Strain sensitivity (Hz/µε) as a function of film thickness (nm) under both compressive (black line) and tensile forces (red line).

The graph in Figure 6 shows how the strain sensitivity of the double-clamped beam (expressed in Hz/µstrain) varies as a function of thickness under both compressive (black line) and tensile forces (red line). The strain sensitivity decreases with thickness (up to ~650 nm) for both compressive and tensile forces. This suggests that thinner structures or those with lower residual stress are more sensitive to both types of forces up to this point. Both curves reach a minimum sensitivity at around 650 nm. This indicates that very thin layers with low intrinsic residual stress have the highest strain sensitivity. Tensile sensitivity is consistently higher than compressive sensitivity for each thickness. This means the structure is more responsive to stretching than compression, possibly due to the material characteristics of the tensile strain. The anisotropic model used in the simulation appears to accurately capture the strain sensitivity trends across all samples.

**Figure 7:**
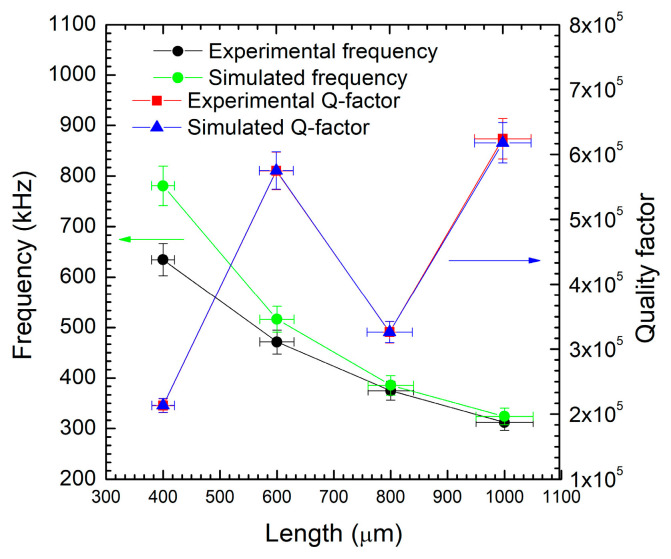
Frequency trends (kHz), both experimental (black line) and simulated (green line), and Q-Factor values, both experimental (red line) and simulated (blue line), as a function of the length (μm).

In Figure 7, we have shown that for a fixed thickness of 890 nm ($w_{1}$), both experimental and simulated frequencies (black and light green, respectively) decrease as length increases. This trend aligns with typical mechanical resonance behavior, where longer structures resonate at lower frequencies. Simulated frequencies are consistently higher than the experimental ones, which may result from ideal conditions assumed in the simulations (i.e., no damping, perfect material properties). The study and the consideration of defect density have already been reported in previous work [3] in which a bilayer model was developed. The 3C-SiC film can be considered a double layer with rich defect density near the interface film/substrate and a high-quality layer above the rich defect, with a reduction of them but not a total elimination. The experimental Q-Factor values (red line) have small fluctuations across most lengths, and the simulated Q-Factor curve (blue line) follows a similar trend because it has been fitted on the experimental values. This suggests that 600 nm is an optimal length for maximizing the Q-Factor, which is crucial for sensing performance and energy efficiency in resonant systems. But, a discrepancy between the simulation and the experiment can be found; while trends are similar, simulated values for frequency are generally higher than the experimental ones.

This discrepancy may arise due to real-world losses (i.e., air damping, fabrication imperfections) not accounted for in the simulations. The figure shows a clear inverse relationship between length and frequency and highlights 600 µm as the optimal length for high Q-Factor performance, both experimentally and in simulations. Despite some quantitative differences, simulation and experimental results are qualitatively consistent, reinforcing the validity of the model and the usefulness of this length for device optimization.

**Figure 8:**
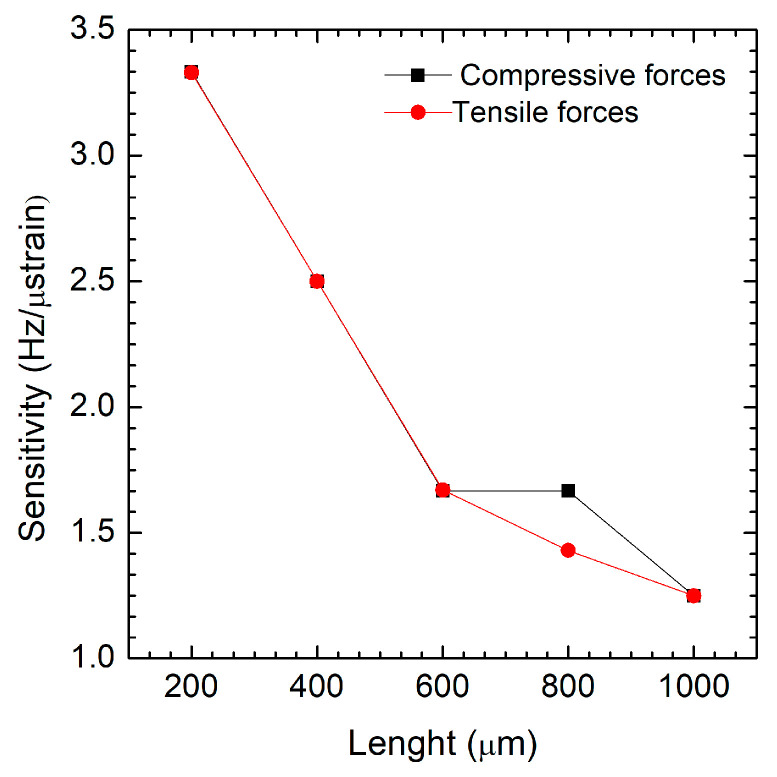
Strain sensitivity (Hz/µε) as a function of film length under both compressive (black line) and tensile forces (red line).

In Figure 8, for both compressive and tensile forces, strain sensitivity decreases as the length increases for a fixed thickness of 890 nm. This trend suggests that shorter structures are more sensitive to external mechanical loading. In a comparison between compressive and tensile forces, the compressive force sensitivity is slightly higher than compressive force sensitivity across all lengths, which is especially notable at intermediate values (e.g., ~700–800 µm). This may indicate that the device responds more effectively (in terms of frequency change) under compressive loading. The figure demonstrates that device length is a key factor influencing frequency-based sensitivity, with shorter devices offering higher sensitivity. Additionally, the system appears to be slightly more responsive under compressive stress, although the difference is relatively small. This information can be useful when optimizing device geometry for strain sensing applications where high frequency sensitivity is critical.

## 4. Discussion

The results of this study make clear the need to include anisotropic damping models in the finite element simulation of 3C-SiC MEMS resonators. Especially for thicker films, better agreement between experimental and simulated resonance frequencies supports the idea that isotropic models cannot adequately represent the directional nature of energy loss in these substances. This finding is consistent with Romero et al.’s (2020) [6] evidence showing that to precisely simulate laminate structures, engineering the dissipation in crystalline micromechanical resonators calls for taking anisotropic loss mechanisms into account in FEM models. Similarly, Somesan and Barti (2020) [2] underlined the need for FEM models with transverse isotropic damping features. These findings support the approach used in this study, whereby COMSOL Multiphysics was presented with anisotropic damping utilizing a 6 × 6 symmetric loss factor matrix. Furthermore, the mechanical behavior of 3C-SiC beams reported here is consistent with Thomsen’s (2014) [7] and Yang et al.’s (2020) [8] findings on the anisotropic elastic properties of silicon and silicon carbide, which emphasize a pronounced stiffness dependence in these materials. The eigenvalue analysis of the damping matrix conducted in this study also supports the claim that energy dissipation is directional and therefore anisotropic modeling is required. The strain sensitivity patterns seen under both compressive and tensile loading also support earlier conclusions. For example, the research by Flis et al. (2025) [9] showed that the precision of FEM simulations might be much impacted by damping coefficients derived from MEMS accelerometers. Showing how strain sensitivity changes with both thickness and length, the current study adds to this knowledge and offers insightful information for the creation of high-performance strain sensors.

Finally, the experimental approach used in this study supports the use of MEMS platforms for mechanical characterization, as discussed by NCSU (2023) [12]. Integrating experimental data with simulations helps one fully grasp the mechanical and damping characteristics of 3C-SiC MEMS devices, therefore guiding the development of more precise and dependable sensor design.

## 5. Conclusions

In conclusion, by using the anisotropic loss factor, a better prediction of the frequency response of the bridge structures has been made, especially at higher thickness. Through this model, both compressive and tensile forces have been applied to the structures, and it has been observed that higher strain sensitivity can be obtained for thinner layers and for shorter line lengths. The higher sensitivity of the thinner layers is probably related to the fact that these layers present a lower residual stress. The work shows that adding directionally dependent damping models to finite-element runs greatly improves how well those runs predict the dynamic response of tiny mechanical components, with 3C-SiC double-clamped beams serving as a key example. Unlike standard isotropic treatments, the new orientation-specific loss factor matrix captures energy loss linked to crystal direction and beam shape, allowing simulations to mirror reality far more closely. Data show that the anisotropic approach matches experimental measurements more closely, especially when estimating resonance frequencies for beams of different film thicknesses. The gap is largest for thicker layers over 600 nm, where the isotropic scheme misses frequency trends and produces unrealistically large response amplitudes. Eigenvalue analysis of the loss factor map points to the directional nature of damping: energy does not leak out evenly but rather in paths set by the materials’ own anisotropy. The findings also stress that overall geometry—thickness, length, and the like—governs how much strain under load, whether pushing or pulling, actually feels like stress. Short, slender samples twist more easily, so their damping rate spikes; in contrast, hefty beams hold residual stress longer and bleed energy slowly. That pattern holds in both tensile and compressive tests, though the tensile case tends to produce sharper rises in sensitivity.

The indications reported in this work can be extremely useful to realize high-sensitivity strain meters.

## Figures and Tables

**Table 1:** Wafer ID and film thickness of samples; specifically, wafers $w_{4}$ and $w_{5}$ refer to the samples used in the Romero at al. paper [6].

**Wafer ID**	$w_{1}$	$w_{2}$	$w_{3}$	$w_{4}$	$w_{5}$
**Thickness (nm)**	890	730	610	337	293

**Table 2:** Table of the eigenvalues and related directions and damping.

Eigenvalue	Direction	Damping
$a_{11}$	*x*	Direction *x*
$a_{22}$	*y*	Direction *y*
$a_{33}$	*z*	Direction *z*
$a_{44}$	*yz*	Direction *yz* plane
$a_{55}$	*xz*	Direction *xz* plane

**Table 3:** Table of percentage errors between simulated and experimental values for each sample.

ID Sample	Compressive	Tensile	Error (%)
$w_{1}$	0.03	0.028	6.67
$w_{2}$	0.04	0.038	5
$w_{3}$	0.05	0.048	4
$w_{4}$	0.08	0.075	6.25
$w_{5}$	0.08	0.085	5.56

## Data Availability

Data will be made available upon request.
